# Anti-cancer efficacy of nonthermal plasma dissolved in a liquid, liquid plasma in heterogeneous cancer cells

**DOI:** 10.1038/srep29020

**Published:** 2016-07-01

**Authors:** Ngoc Hoan Nguyen, Hyung Jun Park, Sang Sik Yang, Kyeong Sook Choi, Jong-Soo Lee

**Affiliations:** 1 Department of Life Sciences, Ajou University, Suwon, Korea; 2 Department of Electrical and Computer Engineering, Ajou University, Suwon, Korea; 3 Ajou University School of Medicine, Suwon, Korea

## Abstract

The therapeutic potential of nonthermal plasma for cancer treatment has been reported recently. The heterogeneity of cancer cells need to be addressed to design effective anticancer treatments. Here, we show that treatment with nonthermal atmospheric-pressure plasma dissolved in a liquid (liquid plasma) induces oxidative stress in heterogeneous populations of cancer cells and ultimately kills these cells via apoptosis, regardless of genetic status, e.g., mutations in p53 and other DNA-damage-response genes. We found that liquid plasma markedly increased the concentration of intracellular and mitochondrial reactive oxygen species (ROS), reflecting an influx from the extracellular milieu. Liquid plasma contributed to mitochondrial accumulation of ROS and depolarization of mitochondrial membrane potential with consequent cell death. Healthy normal cells, however, were hardly affected by the liquid-plasma treatment. The antioxidant N-acetylcysteine blocked liquid-plasma-induced cell death. A knockdown of CuZn-superoxide dismutase or Mn-SOD enhanced the plasma-induced cell death, whereas expression of exogenous CuZn-SOD, Mn-SOD, or catalase blocked the cell death. These results suggest that the mitochondrial dysfunction mediated by ROS production is a key contributor to liquid-plasma-induced apoptotic cell death, regardless of genetic variation. Thus, liquid plasma may have clinical applications, e.g., the development of therapeutic strategies and prevention of disease progression despite tumor heterogeneity.

Extensive morphological, functional, and phenotypic heterogeneity arises among cancer cells within the same tumor and between primary tumors and metastases as a consequence of genetic variation, environmental differences, and epigenetic changes. In tumors, dynamic genetic variations in the course of tumorigenesis can give rise to genetically distinct subpopulations of cancer cells and thereby may affect survival, proliferation, and resistance to treatment among cancer cell subpopulations^1^. Furthermore, intermingled heterogeneous subpopulations are observed within a single biopsy and respond differentially to treatment. Therefore, the tumor heterogeneity originating from this genetic variation is an obstacle to effective cancer treatment and diagnosis and may necessitate personalized treatment. The heterogeneity of cancer cell populations poses substantial challenges to the design of effective strategies for both diagnosis and prognosis.

Genetic heterogeneity is a common feature of cancer cell populations and can arise from multiple sources, thus generating genetically distinct subpopulations that can show differential survival, proliferation, and therapeutic responses^2^. A major source of genetic heterogeneity in cancer is genomic instability, which can arise via various mechanisms and often develops when key regulatory pathways are impaired. For example, disruption of DNA damage responses (DDRs) including DNA repair pathways and DNA damage checkpoint mechanisms can lead to instability of genome structure by promoting replication or correction errors. Furthermore, ongoing large-scale gain or loss of chromosomes in dividing cancer cells has been ascribed to defects in the mitosis machinery or mitotic checkpoint pathways. Genomic instability in the structure and number of chromosomes can develop during tumorigenesis and progression and differentially affects drug sensitivity and patients’ outcomes.

Genomic instability, however, can also be a tempting therapeutic target. Generally, defects in the DDR, including DNA repair and checkpoints, have been utilized for the treatment of cancer with radiation therapy or genotoxic chemotherapy^3^. The cellular response to DNA damage is either survival via DNA damage repair or cell death. Consequently, the DNA damage repair capacity of cancer cells has a major influence on the effectiveness of genomic-instability-targeting therapies involving genotoxic chemicals or radiation. DNA damage activates DNA damage signaling pathways and induces cell cycle arrest, which gives the cell time to repair the damaged DNA. Radiation or genotoxic drugs, which cause DNA damage—that exceeds the repair capacity and leads to death of cancer cells—have been the mainstay of cancer treatment for over 30 years. On the other hand, a tumor’s resistance to genotoxic radiation or chemotherapy can result from increased activity of DNA damage repair, evasion of cell death, mutations in the drug target, increased drug efflux, and activation of alternative signaling pathways including checkpoint or survival mechanisms. In addition, tumors are heterogeneous; therefore, resistance can also arise because of positive selection of a drug-resistant or radioresistant subpopulation.

Aside from predisposition to hereditary or sporadic cancers, DDR defects have also been implicated in drug responsiveness^3^,^4^,^5^,^6^. Mutations in a canonical component of the DDR machinery—the p53 tumor suppressor gene—are common among various types of human cancer. A number of studies have clearly shown that p53 induces apoptosis in cells exposed to genotoxic factors, and a mutation in p53 is frequently associated with drug resistance^4^,^5^,^7^,^8^,^9^,^10^. Additionally, defects in another DDR molecule, BRCA1 (a mutation or reduced expression of the BRCA1 protein), via epigenetic downregulation, are associated with breast cancer stem cells in a mouse model and in human cancers^11^,^12^ and result in aggressive clinical course of breast and ovarian tumor^13^. Moreover, most cancers have a defect(s) in at least one repair pathway, and this problem can lead to recruitment of an available alternative repair pathway; therefore, a cancer cell can evade cell death induced by genotoxic therapies. Even if personalized or more specific cancer treatments (that are targeted to each tumor according to the specific genetic defects) can be applied, analysis of the genetic patterns of each tumor is required, and genetic/cellular heterogeneity within the same tumor can differentially affect the therapeutic response and cause resistance. Therefore, a new approach that works despite tumor heterogeneity—with adequate efficacy and acceptable adverse effects—is urgently needed.

Plasma is a gas-like reactive mixture containing ionized and charged particles, electrically neutral particles, and activated radicals. Multifunctional anticancer activities of plasma have been demonstrated recently^14^,^15^,^16^,^17^,^18^,^19^,^20^,^21^,^22^,^23^,^24^. We have previously reported that nonthermal plasma has potent antitumor activity and has little effect on healthy cells^17^,^18^,^19^,^20^. In a mechanistic extension of that study, we showed that nonthermal plasma generates various radicals, including ROS and/or reactive nitrogen species (RNS), thus causing mitochondrial dysfunction by disrupting mitochondrial membrane potential and ROS accumulation and thereby leading to death of the cancer cell via apoptosis^17^,^19^. These effects of nonthermal-plasma-induced mitochondrial dysfunction prompted us to evaluate the suitability of plasma as a treatment option that can solve the problem of tumor heterogeneity. Here, we show that treatment with nonthermal plasma dissolved in a liquid (liquid plasma) causes apoptosis in heterogeneous populations of DDR-defective cancer cells harboring a p53 deficiency or mutations, an ATM or BRCA1 deficiency, either in single culture or in coculture with healthy cells or other heterogeneous cancer cells. We also found that plasma’s effects are specific to cancer cells. Our results confirm that plasma induces extensive mitochondrial dysfunction in heterogeneous populations of cancer cells by stimulating production of ROS or RNS. Antioxidants or overexpression of ROS-scavenging enzymes, such as Cu- or Mn-superoxide dismutase and catalase, blocked the plasma-induced death of cancer cells and abrogated the mitochondrial aberrations, suggesting that oxidative targeting of mitochondria by plasma precedes selective death of cancer cells. Furthermore, in this study, nonthermal plasma that is dissolved in a liquid was tested for anticancer effects to address the problem of limited penetrance, which is an obstacle to broad adoption of this approach and to effective delivery to tumors that vary in location from external (such as skin cancer) to internal tissues (a tumor inside the body). Taken together, our results suggest that research on plasma medicine may lead to the development of a novel anticancer treatment that is selectively cytotoxic to tumor cells (by targeting mitochondria) and is effective despite genetic heterogeneity within and between tumors.

## Results

### Liquid plasma exerts potent anticancer effects

**Figure 1:**
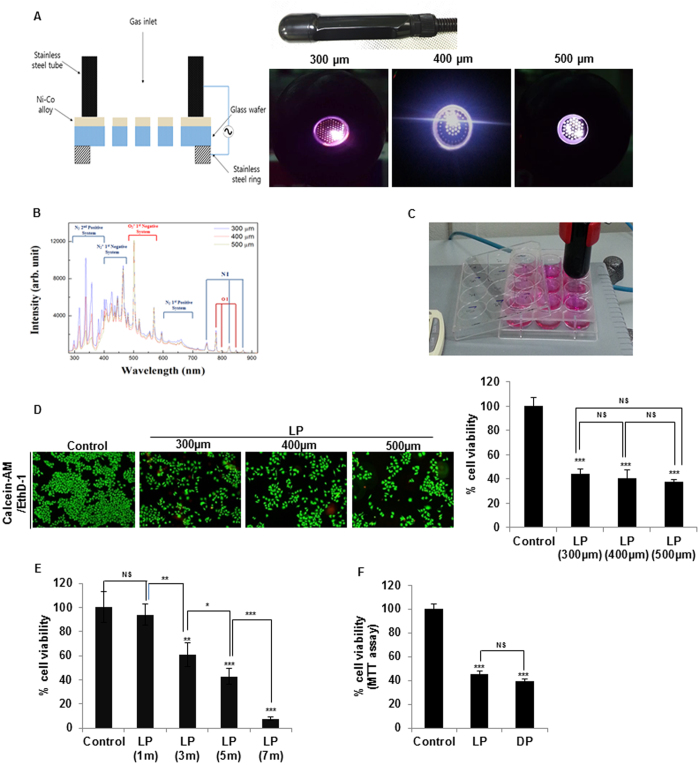
Preparation of liquid plasma (LP) using a microplasma system and the effects on cancer cells. (**A**) A photo of the hand-held-type system that is a microplasma jet device, and photographs of the air microplasma jet generated at atmospheric pressure by means of a nozzle with pores 300–500 μm in diameter. (**B**) Optical emission spectra of air microplasma jets during discharge in the wavelength range 280–920 nm with the end diameters of the collimated plasma jets from 300 to 500 μm. (**C**) Preparation of water plasma via exposure of a medium to plasma located at a ~2-cm distance. Media (DMEM or RPMI 1640) supplemented with 1% fetal bovine serum (FBS) were added to wells of a 12-well-plate (1 mL per well), and then exposed to the plasma jet for indicated periods. (**D**) Viability of HeLa cells was assessed by the live/dead assay after treatment of the cells with 5 min LP generated by means of 300-, 400-, or 500-μm collimated plasma jets. Representative images of calcein-AM/EthD-1 - stained cells under a fluorescence microscope are shown. The right graph shows static analysis of viability of LP-treated HeLa cells compared to untreated (control) cells. (**E**) HeLa cells were treated with liquid plasma generated by air plasma spraying above the media surface for the indicated times (1, 3, 5, and 7 min), and Live/dead assay was performed as described in Materials and Methods. (**F**) Comparison of HeLa cell viability by the MTT assay after 24 h of treatment with liquid or direct plasma. NS, not significant; *P ≤ 0.05, **P ≤ 0.01, ***P ≤ 0.001.

Previously, gas plasma that was directly blown on cancer cells (direct plasma) has been shown to selectively kill cancer cells^17^,^18^,^19^,^20^. To overcome the problem of limited penetrance of plasma (a drawback vis-à-vis the efficacy and specificity to cancer cells), we examined the anticancer effects of nonthermal (cold) air plasma dissolved in a liquid (liquid plasma). To this end, we first prepared liquid plasma (Fig. 1) by means of a microplasma jet system operated at atmospheric pressure^17^,^18^,^19^,^21^ (Fig. 1A) that yielded the optical emission spectra (OES) associated with ROS or RNS (Fig. 1B). Liquid plasma was produced by blowing a jet of cold gas plasma generated at atmospheric pressure onto a liquid such as a mammalian cell culture medium called Dulbecco’s modified Eagle’s medium (DMEM) or Roswell Park Memorial Institute medium (RPMI 1640; Fig. 1C). Then, this liquid containing plasma was used against cancer cells. The OES of cold air plasma generated by means of nozzles of various pore sizes (300, 400, or 500 μm in diameter) were analyzed to identify chemically active oxygen and nitrogen species generated by cold air plasma (Fig. 1A,B). Overall, OES patterns were similar for three cold air plasma jets at different pore sizes of the nozzles. The dominant oxygen emission lines were identified as excited oxygen ions (O_2_^+^) at 500~600 nm and atomic oxygen (O I) at 777 and 844 nm. Additionally, we detected RNS: excited nitrogen molecules (N_2_ second positive system and N_2_ first positive system) in the ranges 300~390 and 610~710 nm and ionized nitrogen molecules (N_2_^+^ first negative system) in the range 390–480 nm, and atomic nitrogen (N I) at 747, 822, and 868 nm (Fig. 1B).

Next, to evaluate the anticancer effects of liquid plasma, we incubated HeLa cells with liquid plasma (prepared at different pore sizes of the nozzle) for 24 h, and stained the cells with calcein AM and EthD-1 to differentiate live and dead cells, respectively (live/dead assay; Fig. 1D). The percentage of live cells was assessed by counting the cells with exclusively green fluorescence, excluding bicolored (green and red) cells. All types of liquid plasma (prepared by means of nozzles of various pore sizes) reduced the viability of HeLa cells to approximately 40% (Fig. 1D), in agreement with their similar OES patterns. Thus, we used the liquid plasma generated from a collimated plasma jet 400 μm in diameter in subsequent experiments here. Next, we investigated dose effect of liquid plasmas differing in exposure time to the air plasma spray for 1 to 7 minutes. Treatment of HeLa cells with liquid plasma exposed to the air plasma spray for 1 min barely affected cell death (Fig. 1E). In contrast, treatment with liquid plasma exposed for 3 min increased cell death by approximately 39.1%. Higher cell death effects of liquid plasmas exposed for 5 (approximately 57.1%) and 7 min (approximately 92.4%) were achieved. These data suggest that liquid plasma induces cancer cell death in a dose-dependent manner (Fig. 1E). Taken together, these results indicated that liquid plasma had anticancer effects similar to the effects of direct treatment of HeLa cells with cold air plasma^17^,^18^,^19^, wherein cancer cells were directly exposed to jets of cold air plasma (Fig. 1F).

**Figure 2:**
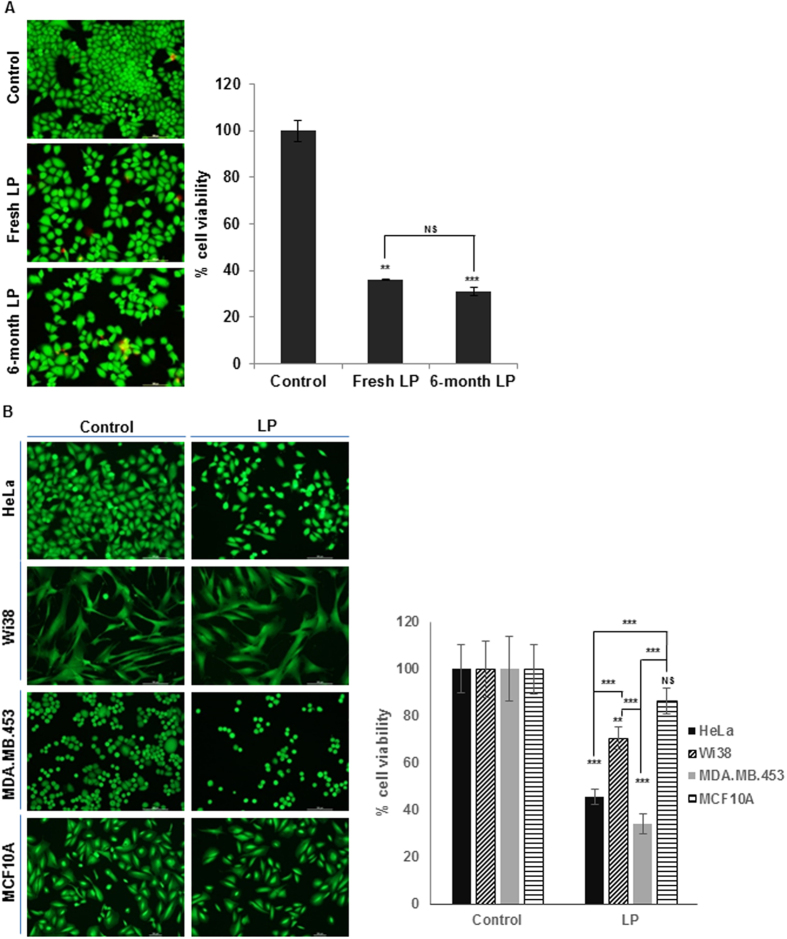
Efficacy of frozen and long-term stored liquid plasma (LP) and its selective anticancer effect. (**A**) HeLa cells were treated with frozen-and-thawed LP that was kept in a freezer (−20°C) for up to 6 months and Live/Dead assay was performed. Viable cells were identified under a fluorescence microscope (Nikon Inverted Microscope Eclipse Ti-S/L100) and counted. (**B**) LP were added to cancer (HeLa, MDA-MB-453) and normal (Wi38, MCF10A) cells, and cell viability was assessed by live/dead assay after 24 h incubation. NS, not significant; *P ≤ 0.05, **P ≤ 0.01, ***P ≤ 0.001.

To use liquid plasma as medicine for therapeutic applications, desired therapeutic potency for a certain period has to be sustained. Thus, we next evaluated the anticancer efficacy of liquid plasma as a function of storage duration. We compared the anticancer effects of freshly prepared liquid plasma with the anticancer activity of liquid plasma stored at −20 °C for 1 to 6 months. The live/dead assay (Fig. 2A) and MTT assay (Supplementary Fig. S1A) of the HeLa cells treated with stored or freshly prepared liquid plasma yielded similar results on cell death. In addition, the liquid plasma kept at room temperature for 1 month had quite similar anticancer effects; even repeated opening and closing of the storage tubes barely affected the anticancer effects (Supplementary Fig. S1B).

Based on the previous reports that air plasma exhibits cancer-selective cytotoxicity^17^,^18^,^19^,^20^, we next tested cytotoxicity of liquid plasma on two human normal cell lines, WI38 (lung fibroblast) and MCF10A (breast epithelial cells) and two cancer cell lines, HeLa (cervical) MDA-MB-453 (breast). Liquid plasma killed cancer cells by approximately 55% to 66% but only by approximately 30% in WI38 cells and 13% in MCF10A cells (Fig. 2B), suggesting that liquid plasma induces cell death more efficiently in cancer cells than in normal cells. Collectively, these results indicate acceptable difference between desired therapeutic effects of liquid plasma on cancer cells and adverse cytotoxic effects on normal cells, consistent with the previous reports^17^,^18^,^19^,^20^.

### Liquid plasma exerts anticancer action on different cancer cells

Tumor heterogeneity is a major problem for cancer treatments because it limits the effectiveness of current chemotherapeutic agents or radiotherapy and thus may contribute to recurrence and metastasis. We examined the sensitivity to liquid plasma among different cancer cell lines harboring various mutations related to drug responsiveness including p53^4^,^5^,^7^,^8^,^9^,^10^,^25^, BRCA1^6^, ATM^4^, and CHK2^4^ mutations.

**Figure 3:**
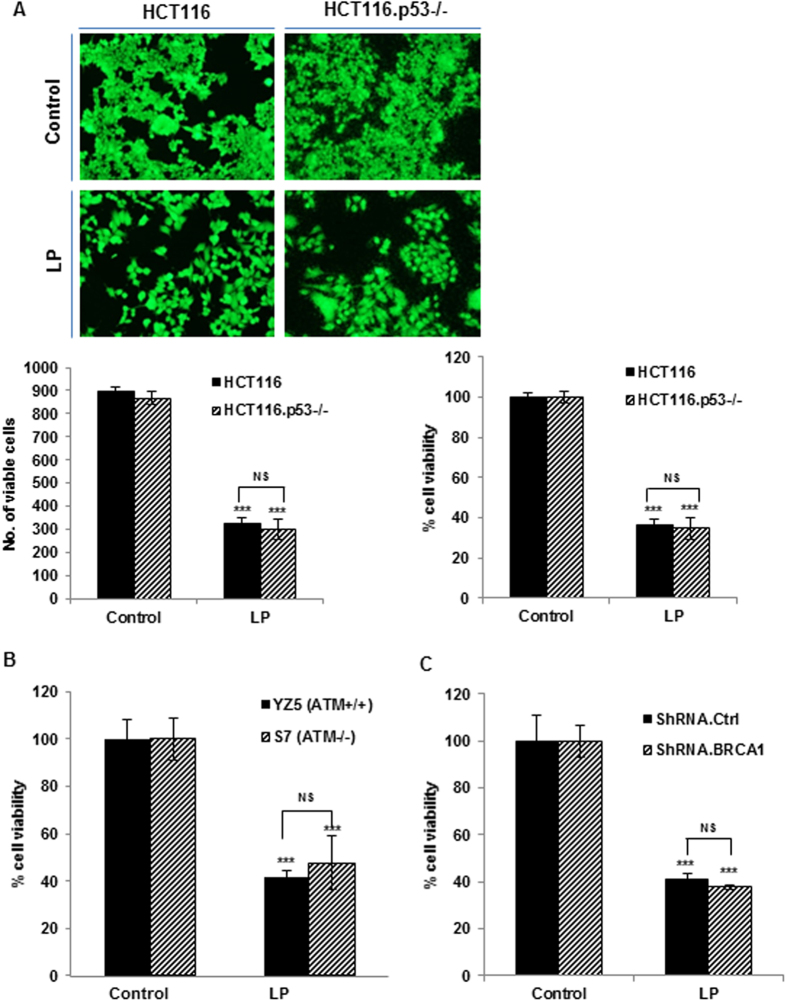
Liquid plasma (LP) induces cell death irrespective of phenotypic and genetic variations. Cancer cells with different genetic alterations were co-cultured using co-culture insert system and treated with 5 min-LP, and cell viability was assessed after 24 h of treatment, by the live/dead assay. (**A**) HCT116 wild-type and HCT116 p53^−/−^ cells; (**B**) YZ5 (ATM^+/+^) and S7 (ATM^−/−^) cells; (**C**) HeLa wild-type cells and HeLa cells with a BRCA1 knockdown by means of short hairpin RNA (shBRCA1). NS, not significant; *P ≤ 0.05, **P ≤ 0.01, ***P ≤ 0.001.

It is well known that tumors with p53 dysfunction, which results in an apoptosis deficiency, are resistant to DNA-damaging chemotherapy and radiotherapy; thus, radio- or chemotherapeutic effectiveness largely depends on p53 status^5^,^8^. Accordingly, resistance to genotoxic drugs was higher in p53-deficient cells compared with p53-positive cells, in a context of HCT116 isogenic colorectal cancer cells^25^. Thus we analyzed coculture of p53-positive and -deficient HCT116 cells - an *in vitro* model of tumor heterogeneity - to determine whether liquid plasma can have anticancer effects on cocultured differing cancer cells (Fig. 3A). Liquid plasma had anticancer effects on both p53-positive and -negative cocultured cells (Fig. 3A). Also, liquid plasma induced cell death in both p53-positive and -deficient HCT116 cells that were separately cultured and treated by liquid plasma (Supplementary Fig. S1C), indicating that liquid-plasma-induced cell death was the same in the coculture system as in the respective culture conditions. We next confirmed that liquid plasma induced the p53-independent cell death in SaOS2 and U2OS cells, which have different p53 status (Supplementary Fig. S1D). Next, we tested whether a deficiency in a p53 target gene, p21 (which inhibits the G_1_-S transition and promotes cell death), affects the liquid-plasma-induced cell death (Supplementary Fig. S1E). As with p53, the p21 deficiency had no effect on the cell death by liquid plasma.

We further explored the effects of a defective DNA damage response pathway (which is one of the hallmarks of cancer) on the sensitivity to liquid plasma, since ATM-CHK2 pathway is known to be required for the p53-dependent apoptosis induced by radiation^4^,^26^ and loss of ATM-CHK2 axis potentiate glioma radiation resistance^27^. For this purpose, we used ATM-positive cells (YZ5) and -negative cells (S7), and CHK2-positive and -negative HCT15 cells (Fig. 3B, Supplementary Fig. S1F,G). Liquid plasma induced cell death regardless of the status of the DNA damage-responsive ATM-CHK2 signaling pathway. In addition, liquid plasma had a comparably potent lethal effect on cells harboring dominant negative kinase-deficient CHK2. Since BRCAness, i.e. BRCA1 inactivation (both genetic and epigenetic) can be useful as a predictive marker of response to different types of chemotherapy regimens and neoadjuvant therapy in not only heterogeneous breast^28^,^29^ but also in other cancers such as ovarian, prostate, and non-small cell lung cancer^6^, we next tested whether defects in the BRCA1 gene affect the liquid-plasma-induced cell death in BRCA1-positive and BRCA1 knockdown HeLa cells. Liquid plasma treatment caused cell death in a BRCA1-independent manner (Fig. 3C and Supplementary Fig. S1H). As expected, the rates of liquid plasma-induced cell death in the coculture system (which mimics tumor heterogeneity) were comparable to the death rates under the single culture conditions for ATM and BRCA1 positive and negative cells (Fig. 3B,C, Supplementary Fig. S1F and S1H).

We further tested whether liquid plasma induces cell death in heterogeneous triple-negative (estrogen receptor, progesterone receptor, and HER2/neu) breast cancer cells^30^, MDA-MB-453 (Fig. 2B) and MDA-MB-468 and -231 (Supplementary Fig. S1I), for better biological relevance. Liquid plasma treatment induced significant cell death in all tested triple-negative breast cancer cell lines by approximately 59% to 66% (Fig. 2B and Supplementary Fig. S1I), in a similar manner to HeLa cells (approximately 55%), suggesting that heterogeneous triple-negative breast cancers could benefit from liquid plasma treatment. Altogether, these results suggested that liquid plasma caused death among diverse cancer cells.

### Liquid plasma induces apoptosis regardless of p53 mutations

**Figure 4:**
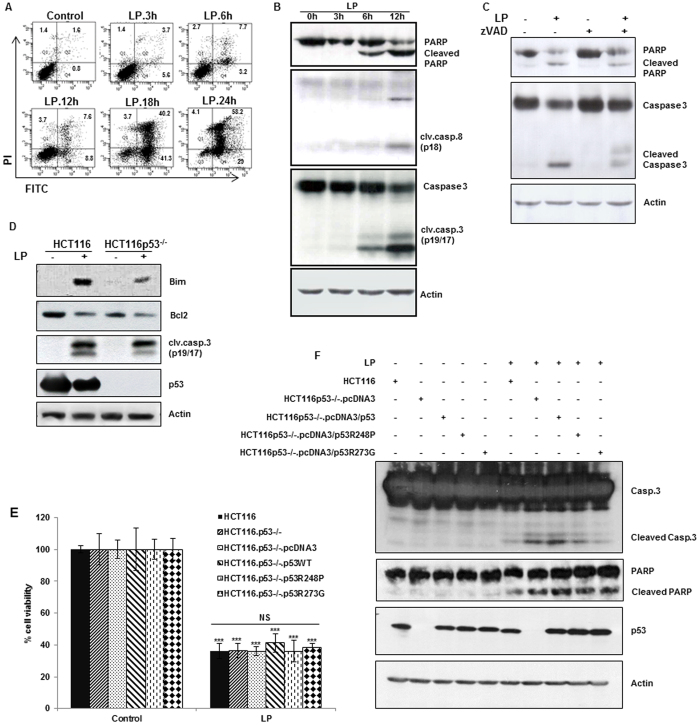
Liquid plasma (LP) induces apoptotic cell death in a p53-independent manner. HeLa cells were treated with LP for the indicated periods. (**A**) Cell death was analyzed by fluorescence-activated cell sorting (FACS) following Annexin V and propidium iodide (PI) staining. (**B**) Activation of the caspase cascade after LP treatment. Immunoblotting was performed with antibodies against cleaved caspase 8 and cleaved caspase 3, PARP, and actin (cropped, full-length images are shown in Supplementary Fig. S4A). (**C**) A caspase inhibitor attenuates LP-induced apoptosis. HeLa cells were treated with z-VAD for 1 h before LP treatment. After 24 h, proteins in cell lysates were separated by sodium dodecyl sulfate polyacrylamide gel electrophoresis (SDS-PAGE) and immunoblotted with antibodies against cleaved caspase 3, PARP, and actin (cropped, full-length images are shown in Supplementary Fig. S4B). (**D**) HCT116 wild-type, and HCT116 p53^−/−^ cells were treated with LP and harvested after 24 h. BH3 protein (proapoptotic Bim) was activated, and prosurvival Bcl-2 was downregulated in response to LP, in a p53-independent manner. Immunoblotting was performed with antibodies against Bim, Bcl-2, caspase 3, p53, and actin (cropped, full-length images are shown in Supplementary Fig. S4C). (**E, F**) Cell viability of HCT116 wild-type, HCT116 p53^−/−^, and HCT116 p53^−/−^ cells with reconstituted p53 (wild type or mutants) was measured by the live/dead assay (**E**) and cellular apoptosis was evaluated by western blot of cleaved caspase 3 and PARP (**F**) after 24 h of LP treatment (cropped, full-length images are shown in Supplementary Fig. S4D). Expression of endogenous or exogenous p53 in these cells was confirmed by western blotting and the amount of actin protein was served as a loading control. NS, not significant; *P ≤ 0.05, **P ≤ 0.01, ***P ≤ 0.001.

To study the mechanisms underlying the cytotoxicity of liquid plasma in a heterogeneous population of cancer cells, first, we used flow cytometry to analyze HeLa cells treated with liquid plasma. The number of early apoptotic (annexin V-positive and propidium iodide-negative) cells increased to 3.2% after 6 h, to 8.8% in 12 h, and to 41.3% in 18 h after liquid-plasma treatment. The number of late apoptotic (annexin V-positive and PI-positive) cells increased to 40.2% in 18 h and to 58.2% in 24 h (Fig. 4A). These data indicated that HeLa cells treated with liquid plasma underwent apoptosis. Thus, we next examined PARP cleavage and the caspase cascade. As canonical markers of apoptosis, cleaved PARP and caspases were detected in liquid-plasma-treated HeLa cells (Fig. 4B). Furthermore, we tested whether a broad-spectrum caspase inhibitor (zVAD) affected the liquid-plasma-induced apoptosis. This inhibitor abrogated the apoptosis and PARP cleavage in the cells treated with liquid plasma (Fig. 4C), confirming that liquid plasma induced death in cancer cells via apoptosis. After that, we confirmed that the liquid-plasma-induced cell death represents apoptosis: we examined the expression of proteins from the Bcl-2 family, which is related to apoptosis. These experiments revealed that proapoptotic Bim was upregulated in response to liquid plasma in both p53-positive and -negative HCT116 cells as well as HeLa cells with silencing BRCA1 (Fig. 4D and Supplementary Fig. S2). In agreement with the upregulation of Bim (which can bind to and inhibit the action of prosurvival members of the Bcl-2 family), Bcl-2 was downregulated (Fig. 4D and Supplementary Fig. S2A). According to these results, liquid plasma induced apoptosis via the Bim-Bcl2 pathway, which is p53 independent.

Next, we tested whether the liquid-plasma-induced apoptosis is dependent on p53 by examining cell death after treatment of p53-positive and -negative HCT116 cancer cells (Supplementary Fig. S3A) with liquid plasma. We confirmed that the p53 status did not affect liquid-plasma-induced cell death, suggesting that p53 is not required for the liquid-plasma-induced apoptosis. Because cancer cells frequently have a p53 mutation, and this type of tumor cells is more malignant than p53-null cells (according to the tumor grade, resistance and recurrence)^31^,^32^,^33^,^34^, we tested whether p53 mutations affect liquid-plasma-induced apoptosis. For this purpose, we used HCT116 isogenic colon cancer cells differing in p53 status: we introduced p53 mutations, which frequently observed in malignant tumors, into p53-negative HCT116 cells and treated these p53-mutant cells with liquid plasma. We assessed the anticancer effects of liquid plasma in the p53-deficient HCT116 cells expressing exogenous wild-type p53 or a mutant (R258P or R273G) p53. Liquid plasma had comparable anticancer effects on all the different cell lines harboring the various p53 variants (Fig. 4E). Additionally, the cleavage of caspase 3 and PARP, which are known markers of apoptosis, was comparably well detectable in all the liquid-plasma-treated p53-deficient HCT116 cells expressing exogenous wild-type or mutant p53 (Fig. 4F). In addition, we confirmed the liquid-plasma-induced cell death was apoptotic: we used the experiment where cell death was inhibited in cancer cells overexpressing anti-apoptotic protein Bcl-xL (Supplementary Fig. S3D). Taken together, these findings suggested that liquid plasma induced apoptosis in a p53-independent manner, and thus liquid plasma can effectively target cancer cells harboring p53 mutations.

### Liquid plasma induces cell death via the mitochondrial apoptotic pathway in an ROS-dependent manner

**Figure 5:**
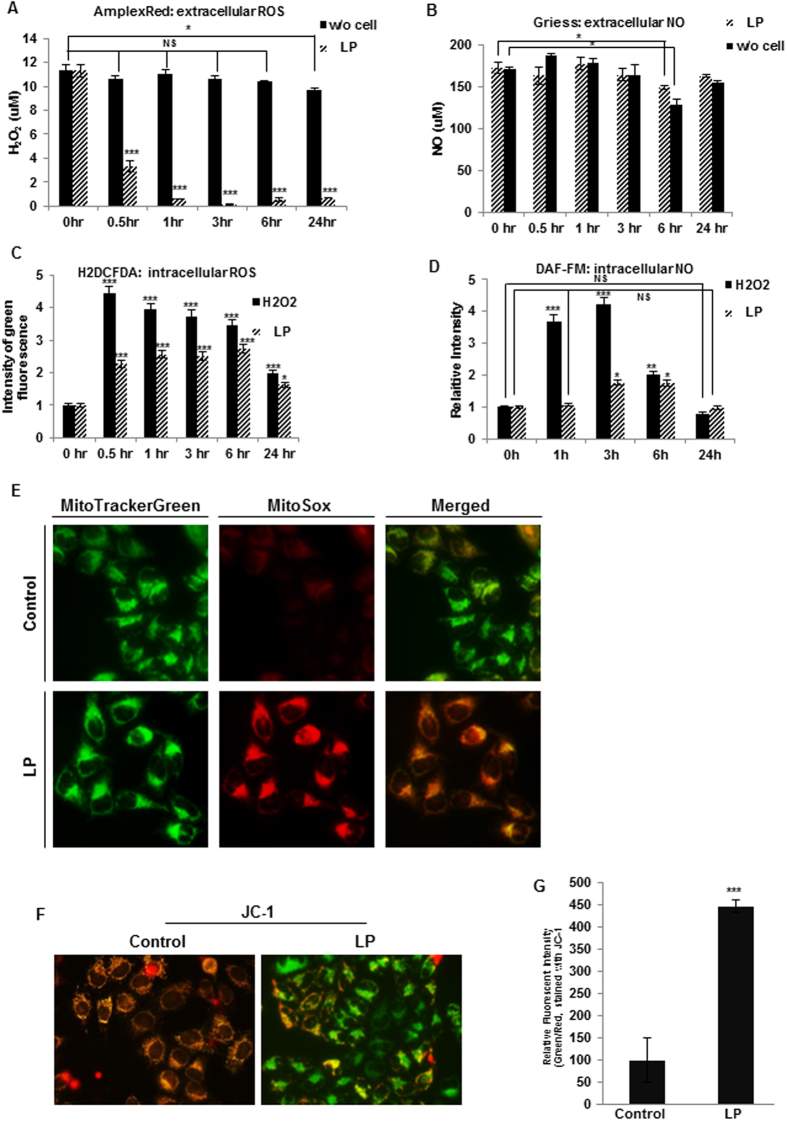
Liquid plasma (LP) generated reactive oxygen and nitrogen species (ROS and RNS), and induced mitochondrial ROS accumulation, resulting in reduced mitochondrial membrane potential (MMP). Extra- and intracellular ROS and RNS were quantified by the AmplexRed assay (extracellular H_2_O_2,_ (**A**)), the Griess assay (extracellular NO, (**B**)), 5,6-carboxy-2′,7′-dichlorofluorescein diacetate (H2DCF-DA) assay (intracellular ROS, (**C**)), and DAF-FM assay (intracellular NO, (**D**)). (**E**) Mitochondrial ROS accumulation was assessed by staining the cells with MitoTracker Green and MitoSox Red. (**F,G**) MMP of HeLa cells was assessed under a fluorescence microscope (F) or measured by fluorescence-activated cell sorting (FACS) (**G**) following 5,5′,6,6′-tetrachloro-1,1′,3,3′-tetraethyl-benzimidazolylcarbocyanine chloride (JC-1) staining. NS, not significant; *P ≤ 0.05, **P ≤ 0.01, ***P ≤ 0.001.

Here, we showed that liquid plasma induced p53-independent apoptosis. Some reports indicate that ROS can induce apoptosis in colon cancer cells in a p53-independent manner^35^,^36^. Previously, direct-gas-plasma-induced cell death was also shown to be accompanied by the production of intra- and extracellular reactive oxygen or nitrogen species^16^,^17^,^18^,^19^,^20^,^37^,^38^ and to involve mitochondrial dysfunction^15^,^17^,^19^,^39^. Because liquid plasma was prepared by blowing gas plasma on a liquid medium, we first determined whether ROS and RNS were generated in liquid plasma. Hydrogen peroxide (H_2_O_2_) and nitrogen oxide (NO) were detected in liquid plasma at concentrations of approximately 11 or 170 μM, respectively, and their levels were maintained for 24 h (Fig. 5A,B, respectively, in HeLa cells and Supplementary Fig. S6A and S6B, respectively, in HCT 116 p53 wild-type and null cells). When the cells were treated with liquid plasma, the level of NO in liquid plasma (extracellular NO level) rarely changed, but H_2_O_2_ concentration rapidly decreased to less than 10% of the initial level. Then, intracellular ROS and NO in the liquid-plasma-treated cells were quantified. The production of intracellular ROS was enhanced approximately two- to three-fold at 0.5–6.0 h after the liquid-plasma treatment, and after 24 h, declined to the basal level (Fig. 5C in HeLa cells and Supplementary Fig. S6C in HCT116 cells). Intracellular NO concentration increased approximately two-fold 3–6 h after the liquid-plasma treatment and decreased back to the basal level in 24 h (HeLa cells, Fig. 5D)

Next, we tested whether liquid plasma was responsible for mitochondrial ROS and affected the mitochondrial membrane potential. Production of ROS in mitochondria under the influence of liquid plasma was detected by means of MitoSox; in contrast, ROS were undetectable in the mitochondria of untreated cells (Fig. 5E). Under a fluorescence microscope, by means of the indicator of mitochondrial membrane potential 5,5′,6,6′-tetrachloro-1,1′,3,3′-tetraethyl-benzimidazolylcarbocyanine chloride (JC-1; Fig. 5F) and by fluorescence-activated cell sorting (FACS) analysis (Fig. 5G), we detected increasing intensity of green/red fluorescence, indicating that liquid plasma can induce a decrease in mitochondrial membrane potential (Δψm) in the liquid plasma-treated cells undergoing death, in contrast to untreated cells. Therefore, these results showed that liquid plasma induced production of mitochondrial ROS/RNS and can lead to mitochondrial dysfunction.

### ROS production under the influence of liquid plasma strongly contributes to liquid-plasma-induced cell death

**Figure 6:**
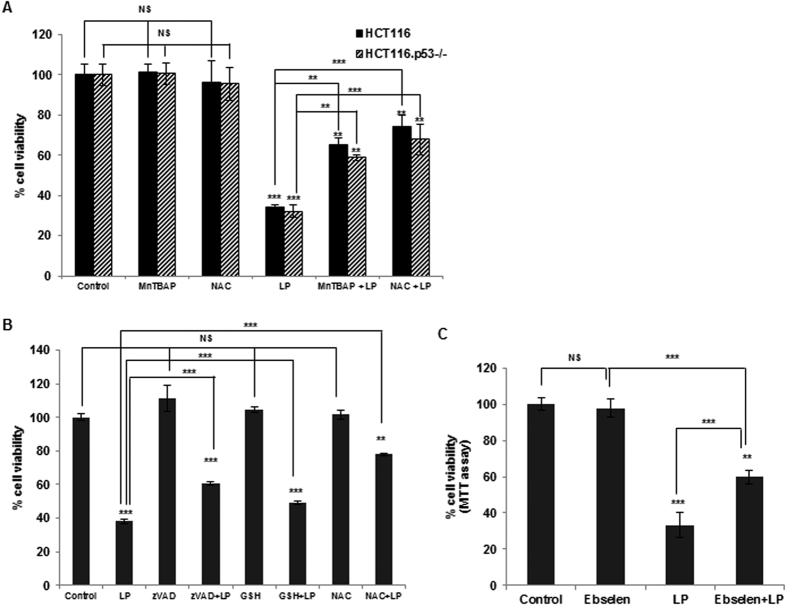
Antioxidants inhibit cell death after treatment with liquid plasma (LP). Antioxidants can strongly inhibit cell death after LP treatment in HCT116 wild-type and HCT116 p53^−/−^ cells (**A**) and in HeLa cells (**B**). Cells were pretreated with MnTBAP, N-acetylcysteine (NAC), reduced glutathione (GSH), or the pan-caspase inhibitor zVAD for 1 h before LP treatment. Cell viability was monitored by the live/dead assay. (**C**) Ebselen - a mimetic of glutathione peroxidase (GPX1) - abrogates HeLa cell death after LP treatment. Ebselen was added to HeLa cells 1 h prior to treatment with LP for 24 h, the cell viability was measured by the 3-(4,5-Dimethylthiazol-2-yl)-2,5-diphenyltetrazolium bromide (MTT) assay. NS, not significant; *P ≤ 0.05, **P ≤ 0.01, ***P ≤ 0.001.

Guided by the results on the ROS and RNS upregulation by liquid plasma (Fig. 5), we tested whether and how liquid-plasma-induced ROS or RNS contributed to cancer cell death. Previously, we have shown that nonthermal gas plasma directly blown on cells causes the production of ROS/RNS during cell death, whereas antioxidants can abrogate the gas plasma-induced death of cancer cells^8^,^11^. Therefore, we determined whether ROS or RNS are involved in liquid-plasma-induced cell death of cancer cells. To this end, we used an SOD mimetic (MnTBAP), a mimetic of glutathione peroxidase (ebselen), reduced glutathione (GSH), and N-acetylcysteine (NAC) as antioxidants to inhibit oxidative stress caused by liquid-plasma-induced ROS/RNS. MnTBAP and ebselen are also known to be peroxynitrite scavengers. All of these scavenging-enzyme mimetics or antioxidants, alone, did not affect cell death (Fig. 6A–C). In contrast, pretreatment of HCT116 WT and HCT116 p53^−/−^ cells or HeLa cells with MnTBAP or ebselen attenuated the liquid-plasma-induced cell death (Fig. 6A,C, respectively). In agreement with these data, the cell death caused by liquid plasma was also attenuated by pretreatment with GSH and NAC (Fig. 6A,B). These results suggested that ROS and RNS generated by liquid plasma are implicated in cancer cell death regardless of p53 status.

**Figure 7:**
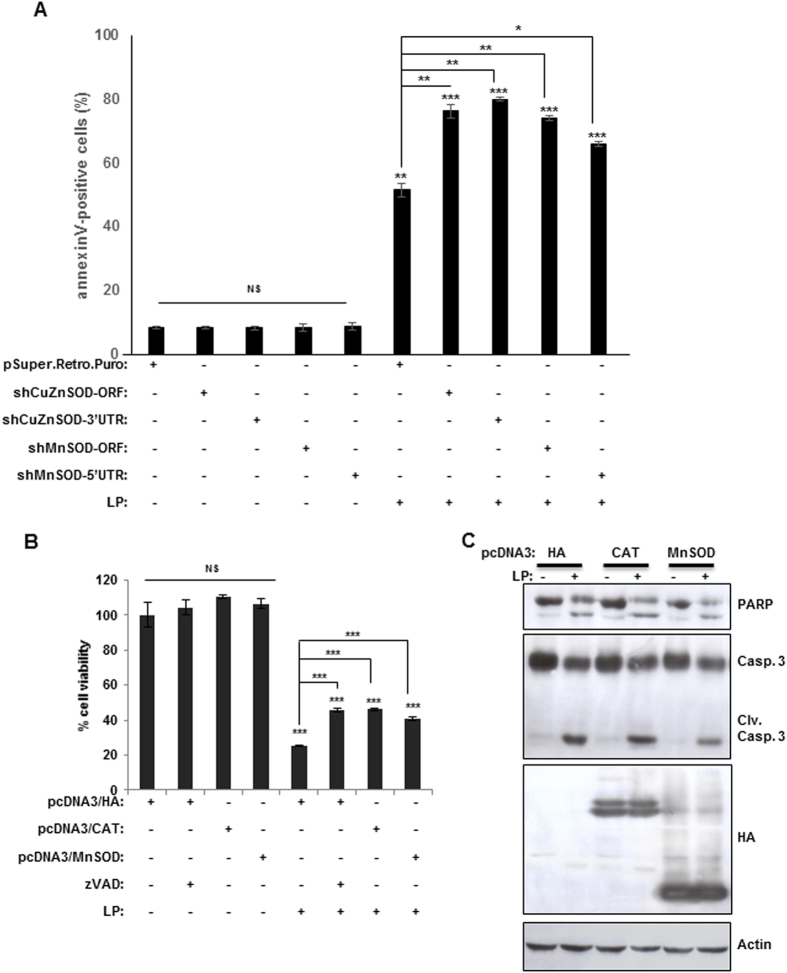
A knockdown of superoxide dismutase (SOD) promotes the LP-induced cell death, and overexpression of this enzyme inhibits LP-induced cell death. HeLa cells were transfected with short hairpin RNA (shRNA) against CuZnSOD or MnSOD (**A**) or the corresponding cDNAs (**B**). The transfected cells were incubated with LP and cell viability was assessed. (**C**) Apoptosis was evaluated by quantification of cleaved caspase 3 and PARP. Immunoblotting with an actin antibody served as a loading control (cropped, full-length blots are shown in Supplementary Fig. S5). NS, not significant; *P ≤ 0.05, **P ≤ 0.01, ***P ≤ 0.001.

To further characterize the liquid-plasma-induced death of cancer cells via ROS or RNS, we determined whether a knockdown or overexpression of the enzymes scavenging reactive species (SOD1/SOD2 and catalase) influenced the liquid-plasma-induced death of cancer cells. The knockdown of either CuZn-SOD (SOD1) or Mn-SOD (SOD2) had no effect on cell viability, but in these knockdown cells, the percentage of cells killed by liquid-plasma increased by approximately 20–30% (Fig. 7A). These data indicated that scavenging of ROS can inhibit the liquid-plasma-induced cell death. To further confirm the role of oxidative radicals in liquid-plasma-induced cell death, we monitored cell death after liquid-plasma treatment of cells overexpressing Mn-SOD or catalase. Exogenously expressed Mn-SOD or catalase inhibited the liquid-plasma-induced cell death (Fig. 7B) and reduced the cleavage of caspase 3 and PARP (Fig. 7C), suggesting that scavenging of ROS by these enzymes can block the liquid-plasma-induced cell death. Collectively, these results showed that liquid plasma upregulates ROS in cancer cells, and ROS is important for the liquid-plasma-induced death of cancer cells.

## Discussion

Heterogeneity of tumor genotypes among patients (interpatient heterogeneity) and within the same patient (intratumor heterogeneity) may render single-target therapeutics ineffective^1^. Tumor heterogeneity driven by genotype variations via random mutations is a serious problem for efficacy of cancer treatment^1^,^2^. A mutation(s) that renders cancer cells resistant to a drug or other treatment may lead to tumor growth, recurrence, metastasis, and treatment failure^1^. Drug resistance related to tumor heterogeneity gives researchers some clues regarding the search for new cancer treatment strategies, and suggests that single-target drugs may not always have the desired effect but multi-target drugs may be more effective against cancer. This is because cancer is a complex disease involving several pathways and multiple genes. Thus, oncologists and cancer biologists have proposed the paradigm of multiple targets or moving targets for drug design or combinational treatments.

This idea is crucial in the context of cancer treatment. A tumor is composed of different tumor cells with distinct genetic and epigenetic profiles resulting in distinct morphological and phenotypic traits including drug sensitivity and malignancy. Moreover, tumor cell populations undergo continuous changes that can confer resistance to a drug; thus, researchers need to target ongoing tumor heterogeneity to solve this ultimate problem. Therefore, the focus of this study was heterogeneous composition of a tumor. In this regard, plasma medicine seems to hold promise as a multitarget therapeutic agent, according to recent reports^14^,^16^,^18^,^19^,^21^,^22^,^23^,^24^,^39^,^40^. Plasma causes DNA damage^21^,^22^,^39^ in a manner similar to that of conventional genotoxic drugs. In addition, plasma can target the cell membrane, possibly via lipid peroxidation^40^ and extracellular matrix^14^, and exerts its effects on DNA^21^,^22^,^39^, JNK/p38^17^,^23^ or NFκB^24^ signaling pathway, and on mitochondria^15^,^16^,^17^,^18^,^39^ in various cancers via production of ROS/RNS as we demonstrated here. Accordingly, plasma can have multiple cellular targets in cancer cells. This multiple-target property of plasma led us to hypothesize its efficacy against tumor heterogeneity. Thus, we explored plasma’s anticancer potential against genotype heterogeneity of a tumor in this work. Notably, liquid plasma inhibited cell growth and induced caspase-dependent apoptosis in heterogeneous cancer cells, especially those harboring p53 mutations correlating with malignancy and aggressiveness. We also tested plasma that is dissolved in a liquid (by blowing gas plasma on a liquid), to try to overcome the limitations of gas plasma, such as low penetrance and accessibility to the internal parts of the body. We show here that liquid plasma is cytotoxic toward various human cancer cell lines that were grown in single culture or in coculture. In contrast to its detrimental effects on cancer cells, liquid plasma showed little or no cytotoxicity toward healthy cells that were grown in single culture or in coculture with cancer cells. Furthermore, two genetically distinct cancer cell lines in coculture (thus mimicking a heterogeneous tumor) were efficiently killed by liquid plasma regardless of genetic variations. Collectively, these results suggest that liquid plasma may be a cancer-specific and multitarget therapeutic agent.

Analysis of the mechanisms underlying the liquid-plasma-induced death of cancer cells revealed that liquid-plasma treatment generates ROS and RNS and sequentially causes apoptosis characterized by pyrokinosis, DNA fragmentation, and caspase activation (i.e., sensitivity to caspase inhibitors). Extra- and intracellular concentrations of radicals and levels of mitochondrial superoxide were markedly increased by liquid plasma, whereas liquid-plasma-induced cell death was inhibited by pretreatment with the antioxidant NAC, GSH, the Mn-SOD-mimetic MnTBAP, or the glutathione peroxidase mimetic ebselen. These data showed the crucial involvement of ROS in the liquid-plasma-induced cell death, and support the radical therapeutic approach by targeting cancer cells by ROS-mediated mechanisms. Increased generation of ROS and an altered redox status in cancer cells have long been exploited for therapeutic benefits. This idea is based on pharmacological ROS insults exceed the ROS limit level in cancer cells but not in normal cells, and thus ROS drugs can preferentially eliminate cancer cells. However, adaptation of cancer cells into intrinsic oxidative tumor microenvironment can confer drug resistance. Thus, studies need to be designed to further address strategies for abrogation of such drug-resistant pathways via adaptation to oxidative tumor microenvironment.

Our group and several other researchers demonstrated the potent anticancer effects of plasma. In addition, plasma has some advantages over conventional chemotherapy and radiotherapy. Plasma as a therapeutic agent can be generated safely and inexpensively. Furthermore, in this work, we showed the specificity of liquid plasma to cancer cells; these findings are in line with those of other reports^14^,^17^,^18^,^19^,^20^,^23^,^24^. Nonetheless, there has been much debate regarding whether plasma is a feasible anticancer agent. The major limiting factor for plasma is its tissue penetrance. Accordingly, another focus of our study was the resolution of this apparent problem with delivery. To overcome this limited internal accessibility, we used liquid plasma in this study. We show that liquid plasma can effectively kill cancer cells, just as gas plasma does. Notably, the storage of liquid plasma was tested for up to 1 year at −20 °C and 4 °C, and the storage tube was opened several times. Liquid plasma can be stored at room temperature for up to 6 months without a significant loss of therapeutic activity.

In summary, we propose liquid plasma as a possible cancer cell-specific multitarget therapeutic agent that can overcome the problem of tumor heterogeneity. ROS generation is responsible for the potent anticancer effects of liquid plasma on heterogeneous populations of cancer cells. Thus, liquid plasma may be a promising anticancer agent whose effects are unaffected by tumor heterogeneity.

## Materials and Methods

### ReaNgents and antibodies

We used the following reagents: NAC, GSH (Sigma Chemical Corp.); MitoTracker Green, calcein acetoxymethyl ester (calcein-AM), ethidium homodimer (EthD-1), 5,6-carboxy-2′,7′-dichlorofluorescein diacetate (H2DCF-DA), MitoSOX red, 5,5′,6,6′-tetrachloro-1,1′,3,3′-tetraethyl-benzimidazolylcarbocyanine chloride (JC-1; Invitrogen); z-VAD-fmk (Promega); MnTBAP (Mn[III] tetrakis[benzoic acid porphyrin]; Calbiochem). We used antibodies against β-actin (Santa Cruz Biotechnology, Inc. and Abcam), Flag-M2 (Sigma), caspase-3, caspase-8 (Cell Signaling Technology), Bcl-2 (Santa Cruz), poly(ADPribose) polymerase (BD Pharmingen), p53 (Cell Signaling Technology and Santa Cruz) and horseradish peroxidase (HRP)-conjugated anti-rabbit or anti-mouse IgG antibodies (Zymed).

### Plasmid construction

The vectors expressing human p53, catalase, MnSOD, or Bcl-xL were generated by inserting the full-length cDNA fragment into pcDNA3-HA (Invitrogen, Carlsbad, CA). Mutant p53 variants R248P and R273G were generated using a QuickChange Site-directed Mutagenesis Kit (Agilent Technologies, Inc., Santa Clara, CA). Double-stranded small interfering RNAs (siRNAs) against BRCA1, SOD1/SOD2, or catalase were generated using a pSUPER.retro.puro, an H1 promoter-driven RNA interference retroviral vector (Oligoengine, Seattle, WA). The siRNAs were designed to target BRCA1 (5′-GATCGACGTGTAGTGAATG-3′), CuZnSOD (ORF, 5′-CAAAGGTGGAAATGAAGAA-3′; 3′UTR, 5′-GCTGTAGAAATGTATCCTGAT-3′), MnSOD (ORF, 5′-GGAGAATGTAACTGA AAGA-3′; 5′UTR, 5′-AGCGGTAGCACCAGCACTA-3′), and catalase (5′-CCAAATACTC CA AGGCAAA-3′).

### Cell culture, transfection, and treatment with liquid plasma

The human cervical carcinoma cell line HeLa as well as sarcoma U2OS and SaOS2 cells and breast cancer cell lines (MDA-MB-231, MDA-MB-453, and MDA-MB-468), and normal cell lines (breast epithelial MCF10A and lung fibroblast WI38) were purchased from the American Type Culture Collection (ATCC), and were cultured in DMEM (WELGENE) supplemented with 10% fetal bovine serum (FBS) and antibiotics (Life Technologies). HCT116 cells (wild type or with a knockout of p53) (gifts from B. Vogelstein, Jonhs Hopkins) were cultured in RPMI 1640 (WELGENE) supplemented with 10% FBS and antibiotics. YZ5 cells (ATM^+/+^) and S7 cells (ATM^−/−^) (gifts from Y. Shiloh) were maintained in DMEM supplemented with 15% FBS and 100 μg/mL hygromycin B. HCT15 Chk2^WT^, HCT15 Chk2^T68A^, and HCT15 Chk2^−/−^ cells (gifts from J Chen, The University of Texas MD Anderson Cancer Institute) were grown in RPMI 1640 supplemented with 10% FBS and antibiotics. All the cells were maintained at 37 °C in a humidified incubator at 5% CO_2_. Transfections were performed with the Effectene Kit (Qiagen, Carlsbad, CA) for expression in mammalian cells. For treatment with liquid plasma, this plasma was produced using a microplasma jet device with pores 300–500 μm in diameter at atmospheric pressure (Fig. 1A). The plasma generation module consists of many components. Its main components are an anode, an insulator, a stainless-steel cathode, and a case. The anode was fabricated by conventional micromachining technology and Ni electroplating. In this process, a Ti/Cu film on a wafer was used as a seed layer for electroplating. The gas was injected through a gas inlet tube and an electric field was applied to two electrode rings. Exposure of a liquid medium to plasma was performed at atmospheric pressure and room temperature and the gas flow rate was maintained at 10 L/min. Liquid plasma was produced by discharging the plasma jet onto a liquid such as a mammalian cell culture medium called DMEM or RPMI 1640 (Fig. 1C). The cells were washed with DPBS (Life Technologies) and were covered with a fresh culture medium that was either not treated or exposed to a microplasma jet for 5 min (liquid plasma). Two cell lines were cocultured in a 6-well plate with inserts (SPL, Life Science) and were treated with liquid plasma.

### Quantification of ROS and RNS

Concentrations of H_2_O_2_ and ROS were measured using the Amplex UltraRed hydrogen peroxide assay kit (Invitrogen) and H2DCFDA, respectively^17^. To quantitate ROS, we incubated cells with 5 μM H2DCF-DA for 30 min. Florescence intensity was measured on a microplate reader (Bio-Rad, Hercules, CA) at 530/590 nm (Amplex UltraRed) or 485/535 nm (H2DCF-DA), according to the manufacturer’s protocol. The relative ROS level was calculated in terms of arbitrary fluorescence units. Production of RNS in the culture supernatant by plasma was detected as described previously^17^. To detect the intracellular production of NO, we incubated the cells with 10 μM DAF-FM at 37 °C for 30 min. The cells were analyzed by flow cytometry (BD FACSAria III, BD Bioscience, San Jose, CA).

### Quantification of the mitochondrial membrane potential (∆*ψ*_m_)

Liquid-plasma-treated cells (approximately 10^5^) were incubated for 12–24 h to measure the mitochondrial membrane potential^19^. After 24-h incubation, the cells were stained with 2.5 μM JC-1 (a lipophilic cationic fluorescence dye with a dual emission wavelength) for 30 min, in order to analyze the mitochondrial membrane potential (Δψm). After staining, the cells were analyzed by flow cytometry at 530 nm and 590 nm. JC-1 accumulates within mitochondria in inverse proportion to Δψm. The data are presented as mean ± SD.

### Measurement of cellular viability

The live/dead assay and the viability assay involved double-labeling of cells with 2 μM calcein AM and 4 μM EthD-1, according to the manufacturer’s instruction (Life Technologies). The calcein AM-positive live cells and EthD-1-positive dead cells were examined under a fluorescence microscope (Nikon Inverted Microscope Eclipse Ti-S/L100) and counted. Only exclusively green cells were counted as live because bicolored (green and red) cells cannot be ambiguously assigned to live or dead groups. The percentage of live cells (Live %) calculated as green cells/(green + red + biocolored), was normalized to that of untreated control cells (100%). In addition, MTT assay was performed according to the manufacturer’s protocol (Sigma) after treatments. Absorption at 570 nm was normalized to that of untreated cells (100%), and the data were expressed as cellular viability % of control cells.

### Detection of apoptosis

Cells (approximately 5×10^4^) were seeded in 12-well plates with 1 mL of DMEM or RPMI 1640 supplemented with 10% of FBS and were grown overnight. After treatment with liquid plasma, the cells were incubated for 24 h at 37 °C in a humidified incubator at 5% CO_2_. The cells were harvested with trypsin-EDTA and rinsed with PBS. To detect plasma-induced apoptosis, we stained the cells with an Alexa488-conjugated anti-annexin V antibody or propidium iodide (Invitrogen, Eugene, OR)^17^,^19^. After the staining, the cells were analyzed by flow cytometry (BD FACSAria III, BD Bioscience, San Jose, CA). Data are shown as the mean ± SD (*n* = 5). PARP cleavage and caspase activation were determined as additional apoptotic markers by immunoblotting using anti-PARP or anti-caspase antibody.

### Immunoblotting

Cells were washed in PBS and lysed in boiling sample buffer (62.5 mM Tris-HCl pH 6.8, 1% SDS, 10% glycerol, and 5% β-mercaptoethanol) for sodium dodecyl sulfate polyacrylamide gel electrophoresis (SDS-PAGE). The lysates were boiled for 5 min, and the proteins were separated by SDS-PAGE and transferred to an Immobilon membrane (Millipore). After nonspecific binding sites were blocked for 1 h by means of 5% skim milk, the membranes were incubated for 2 h with specific antibodies. The membranes were then washed three times with phosphate-buffered saline containing Tween 20 (PBST) and incubated further for 1 h with an HRP-conjugated anti-rabbit, anti-mouse, or anti-goat antibody. Visualization of protein bands was accomplished by means of ECL (Amersham Life Science). Representative results from at least three independent experiments are shown in the figures.

### Statistical analysis

All data were expressed as mean ± standard deviation (SD) of at least three replicates. Student’s *t* test was used for the analysis of significance of differences between datasets. Differences were considered statistically significant at *p* ≤ 0.05 (in figures: *p ≤ 0.05, **p ≤ 0.01, ***p ≤ 0.001).

## Additional Information

**How to cite this article**: Nguyen, N. H. *et al*. Anti-cancer efficacy of nonthermal plasma dissolved in a liquid, liquid plasma in heterogeneous cancer cells. *Sci. Rep*. **6**, 29020; doi: 10.1038/srep29020 (2016).

## Supplementary Material

Supplementary Information

## References

[b1] Bedard P. L., Hansen A. R., Rarain M. J. & Siu L. L. Tumor heterogeneity in the clinic. Nature. 501, 355–364, doi: 10.1038/nature12627 (2013). 24048068 PMC5224525

[b2] Burrell R. A., McGranahan N., Bartek J. & Swanton C. The causes and consequences of genetic heterogeneity in cancer evolution. Nature. 501, 338–345, doi: 10.1038/nature12625 (2013). 24048066

[b3] Surova O. & Zhivotovsky B. Various modes of cell death induced by DNA damage. Oncogene. 32, 3789–3797, doi: 10.1038/onc.2012.556 (2013). 23208502

[b4] Jiang H. . The combined status of ATM and p53 link tumor developemnt with therapeutic response. Genes Dev. 23, 1895–1909, doi: 10.1101/gad.1815309 (2009). 19608766 PMC2725944

[b5] Lowe S. W. . p53 status and the effeicacy of cancer therapy *in vivo*. Science. 266, 807–810 (1994). 7973635 10.1126/science.7973635

[b6] James C. R., Quinn J. E., Mullan P. B., Johnston P. G. & Harkin D. P. BRCA1, a potential predictive biomarker in the treatment of breast cancer. Oncologist. 12, 142–150 (2006). 17296808 10.1634/theoncologist.12-2-142

[b7] Yang B., Eshleman J. R., Berger N. A. & Markowitz S. D. Wild-type p53 protein potentiates cytotoxicity of therapeutic agents in human colon cancer cells. Clin Cancer Res. 2, 1649–1657 (1996). 9816112

[b8] Muller P. A. & Vousden K. H. Mutant p53 in cancer: new functions and therapeutic opportunities. Cancer Cell. 25, 304–317, doi: 10.1016/j.ccr.2014.01.021 (2014). 24651012 PMC3970583

[b9] Polyak K., Xia Y., Zweier J. L., Kinzler K. W. & Vogelstein B. A model for p53-induced apoptosis. Nature. 389, 300–305 (1997). 9305847 10.1038/38525

[b10] Sionov R. V. & Haupt Y. The cellular response to p53: the decision between life and death. Oncogene. 18, 6145–6157 (1999). 10557106 10.1038/sj.onc.1203130

[b11] Wright M. H. . Brca1 breast tumors contain distinct CD44+/CD24− and CD133+ cells with cancer stem cell characteristics. Breast Cancer Res. 10, R10, doi: 10.1186/bcr1855 (2008). 18241344 PMC2374965

[b12] Singh A. & Settleman J. EMT, cancer stem cells and drug resistance: an emerging axis of evil in the war on cancer. Oncogene. 29, 4741–4751, doi: 10.1038/onc.2010.215 (2010). 20531305 PMC3176718

[b13] Song L. L. & Miele L. Cancer stem cells–an old idea that’s new again: implications for the diagnosis and treatment of breast cancer. Expert Opin Biol Ther. 7, 431–438, doi: 10.1517/14712598.7.4.431 (2007). 17373895

[b14] Chang J. W. . Non-termal atmospheric pressure plasma inhibits thyroid papillary cancer cell invasion via cytoskeletal modulation, altered MMP-2/-9/uPA activity. PLoS One. 9, e92198, doi: 10.1371/journal.pone.0092198 (2014). 24667444 PMC3965425

[b15] Panngom K. . Preferential killing of human lung cancer cell lines with mitochondrial dysfunction by nonthermal dielectric barrier discharge plasma. Cell Death Dis. 4, e642, doi: 10.1038/cddis.2013.168 (2013). 23703387 PMC3674375

[b16] Ishaq M. . Atmospheric gas plasma-induced ROS production activates TNF-ASK1 pathway for the induction of melanoma cancer cell apoptosis. Mol Biol Cell. 25, 1523–1531, doi: 10.1091/mbc.E13-10-0590 (2014). 24574456 PMC4004600

[b17] Ahn H. J. . Targeting cancer cells with reactive oxygen and nitrogen species generated by atmospheric-pressure air plasma. PLoS One. 9, e86173, doi: 10.1371/ journal.pone.0086173 (2014). 24465942 10.1371/journal.pone.0086173 PMC3897664

[b18] Kang S. U. . Nonthermal plasma induces head and neck cancer cell death: the potential involvement of mitogen-activated protein kinase-dependent mitochondrial reactive oxygen species. Cell Death Dis. 5, e1056, doi: 10.1038/cddis.2014.33 (2014). 24525732 PMC3944250

[b19] Ahn H. J. . Atmospheric-pressure plasma jet induces apoptosis involving mitochondria via generation of free radicals. PLoS One. 6, e28154, doi: 10.1371/ journal.pone.0028154 (2011). 22140530 10.1371/journal.pone.0028154 PMC3226649

[b20] Gweon B. . Differential responses of human liver cancer and normal cells to atmospheric pressure plasma. Appl Phys Lett. 99, 063701 (2011).

[b21] Kim K. I. . Atmospheric-pressure plasma-jet from micronozzle array and its biological effects on living cells for cancer therapy. Appl Phys Lett. 98, 073701 (2011).

[b22] Chang J. W. . Non-thermal aatmospheric pressure plasma induces apoptosis in oral cavity squamous cell carcinoma: Involvement of DNA-damage-triggering sub-G1 arrest via the ATM/p53 pathway. Arch Biochem Biophys. 545, 133–140, doi: 10.1016/j.abb.2014.01.022 (2014). 24486404

[b23] Lee S. Y. . Nonthermal plasma induces apoptosis in ATC cells: involvement of JNK and p38 MAPK-dependent ROS. Yonsei Med J. 55, 1640–7, doi: 10.3349/ymj.2014.55.6.1640 (2014). 25323903 PMC4205706

[b24] Chang J. W. . Combination of NTP with cetuximab inhibited invasion/migration of cetuximab resistant OSCC cells: Involvement of NF-κB signaling. Sci. Rep. 5, 18208, doi: 10.1038/srep.18208 (2015). 26655729 PMC4677387

[b25] Boyer J. . Characterization of p53 wild-type and null isogenic colorectal cancer cell lines resisttant to 5-fluorouracil, oxaliplatin, and irinotecan. Clin Cancer Res. 10, 2158–67 (2004) 15041737 10.1158/1078-0432.ccr-03-0362

[b26] Hirao A. . Chk2 is a tumor suppressor that regulates apoptosis in both an ATM-dependent and an ATM-independent manner. Mol Cell Biol. 22, 6521–32 (2002) 12192050 10.1128/MCB.22.18.6521-6532.2002 PMC135625

[b27] Squatrito M. . Loss of ATM/Chk2/p53 pathway components accelerates tumor development and contribute to radiation resistance in gliomas. Cancer Cell. 18, 619–29 (2010) 21156285 10.1016/j.ccr.2010.10.034 PMC3818087

[b28] Akashi-Tanaka S. . BRCAness predicts resistance to taxane-containing regimens in triple negative breast cancer during neoadjuvant chemotherapy. Clin Breast Cancer. 15, 80–85, doi: 10.1016/j.clbc.2014.08.003 (2015) 25445419

[b29] Andre F. & Zielinski C. C. Optimal strategies for the treatment of metastatic triple-negative breast cancer with currently approved agents. Ann Oncol 23, vi46–51, doi: 10.1093/annonc/mds195 (2012) 23012302

[b30] Chavez K. J., Garimella S. V. & Lipkowitz S. Triple negative breast cancer cell lines: One tool in the search for better treatment of triple negative breast cancer. Breast Dis, 32, 35–48, doi: 10.3233/BD-2010-0307 (2010) 21778573 PMC3532890

[b31] Muller P. A. J. & Vousden K. H. p53 mutations in cancer. Nature Cell Biol 15, 2–8, doi: 10.1038/ncb2641. (2013) 23263379

[b32] Freed-Pasor W. A. & Prives C. Mutant p53: one name, many proteins. Genes Dev, 26, 1266–86. doi: 10.1101/gad.190678.112. (2012) PMC3387655 22713868

[b33] Rivlin N., Brosh R., Oren M. & Rotter V. Mutations in the p53 tumor suppressor gene: important milestones at the various steps of tumorigenesis. Genes Cancer, 2, 466–74, doi: 10.1177/1947601911408889. (2011) 21779514 PMC3135636

[b34] Goh A. M., Coffill C. R. & Lane D. P. The role of mutant p53 in human cancer. J. Pathol. 223, 116–126, doi: 10.1002/path.2784. (2011) 21125670

[b35] Wang L. . Dihydrotanshinone induces p53-independent but ROS-depedent apoptosis in colon cancer cells. Life Sci. 93, 344–351, doi: 10.1016/j.lfs.2013.07.007 ( 2013). 23871989

[b36] Ferreira de Oliveira J. M. . Sulforaphane induces oxidative stress and death by p53-independent mechanism: Implication of impaired glutathione recycling. PLoS One. 9, e92980, doi: 10.1371/journal.pone.0092980 (2014). 24667842 PMC3965485

[b37] Korshunov S. S., Skulachev V. P. & Starkov A. A. High protonic potenetial actuates a mechanism of production of reactive oxygen species in mitochondria. FEBS Lett. 416, 15–18 (1997). 9369223 10.1016/s0014-5793(97)01159-9

[b38] Vandamme M. . ROS implication in a new antitumor strategy based on non-thermal plasma. Int J Cancer. 130, 2185–2194, doi: 10.1002/ijc.26252 (2012). 21702038

[b39] Kim G. J., Kim W., Kim K. T. & Lee J. K. DNA damage and mitochondria dysfunction in cell apoptosis induced by nonthermal air plasma. Appl Phys Lett. 96, 021502 (2010).

[b40] Kim K. I. . Cellular membrane collapse by atmospheric-pressure plasma jet. Appl Phys Lett. 104, 013701 (2014).

