# Supplementary material for: Anti-cancer efficacy of nonthermal plasma dissolved in a liquid, liquid plasma in heterogeneous cancer cells
Source: Sci Rep. 2016 Jul 1;6:29020. doi: 10.1038/srep29020 (PMC4929502; doi:10.1038/srep29020)
Supplement: Supplementary Information [file srep29020-s1.doc]

**<Supplementary information>**

**Anti-cancer efficacy of nonthermal plasma dissolved in a liquid, liquid plasma in heterogeneous cancer cells**

Nguyen Ngoc Hoan^1^, Hyung Jun Park^2^, Sang Sik Yang^2^, Kyeong Sook Choi^3^, and Jong-Soo Lee^1,*^

^1^Department of Life Sciences, ^2^Department of Electrical and Computer Engineering, Ajou University, Suwon, Korea, ^3^Ajou University School of Medicine, Suwon, Korea

*To whom correspondence should be addressed: Jong-Soo Lee, College of Natural Sciences, Ajou University, San 5 Wonchun-Dong, Yeongtong-Gu, Suwon 443-749, Korea; Tel. +82 31-219-1886; Fax +82 31-219-1615; E-mail [jsjlee@ajou.ac.kr](mailto:jsjlee@ajou.ac.kr)


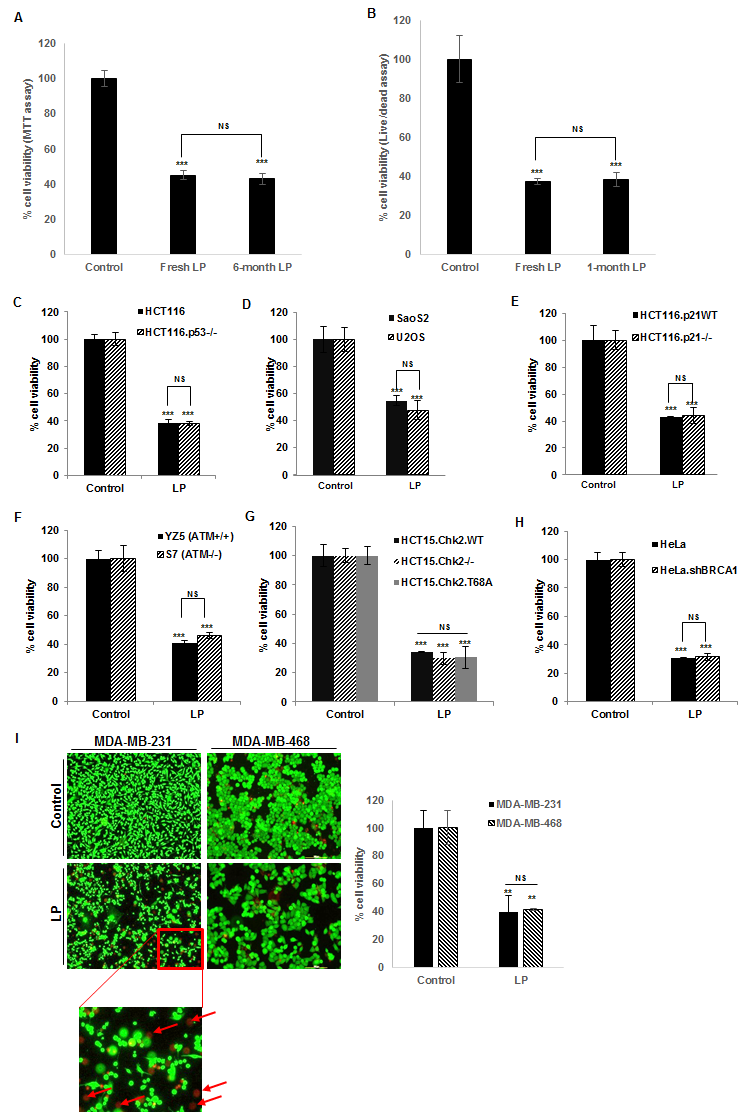


**Figure S1. Efficacy of long-term stored LP and its similar effect in heterogonous cancer cells.** (**A, B**) HeLa cells were treated with frozen-and-thawed LP that was kept in a freezer (−20°C) for up to 6 months (**A**) or at RT for 1 month (**B**). Viable cells were accessed by MTT assay or identified by live/dead assay, respectively. Cancer cells with different genetic alterations were treated with LP, and cell viability was assessed after 24 h of treatment, by the live/dead assay. (**C**) HCT116 wild-type and HCT116 p53^−/−^ cells; (**D**) SaOS2 and U2OS cells; (**E**) HCT116 p21^WT^ and HCT116 p21^−/−^ cells; (**F**) YZ5 (ATM^+/+^) and S7 (ATM^−/−^) cells; (**G**) HCT15 Chk2^WT^, HCT15 Chk2^T68A^, and HCT15 Chk2^−/−^ cells; (**H**) HeLa wild-type cells and HeLa cells with a BRCA1 knockdown by means of short hairpin RNA (shBRCA1); and (**I**) triple negative breast cancer MDA-MB-231 and MDA-MB-468 cells. Dead (red) cells are indicated with arrows in the enlarged view. Box indicates the enlarged region. NS, not significant; **P* ≤ 0.05, ***P* ≤ 0.01, ****P* ≤ 0.001.

**
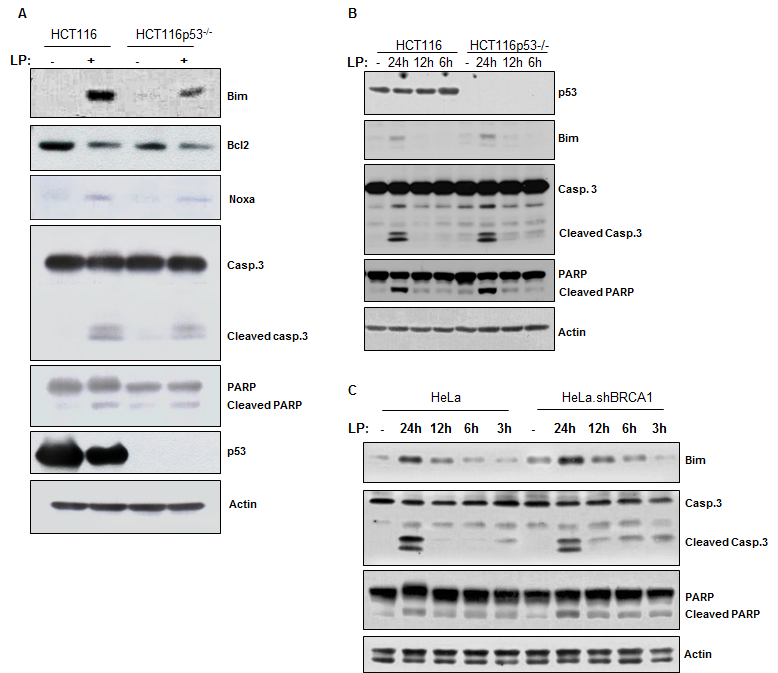
**

**Figure S2. Liquid plasma induces apoptotic cell death in a p53- or BRCA1-independent manner.** p53 wild-type HCT116 or p53^-/-^ HCT116 (**A, B**) and BRCA1 wild-type HeLa or BRCA1-knockdowned HeLa (**C**) cells were treated with LP for the indicated times (24h in **S2A**). Immunoblotting was performed with antibodies against apoptotic proteins and actin as a loading control.


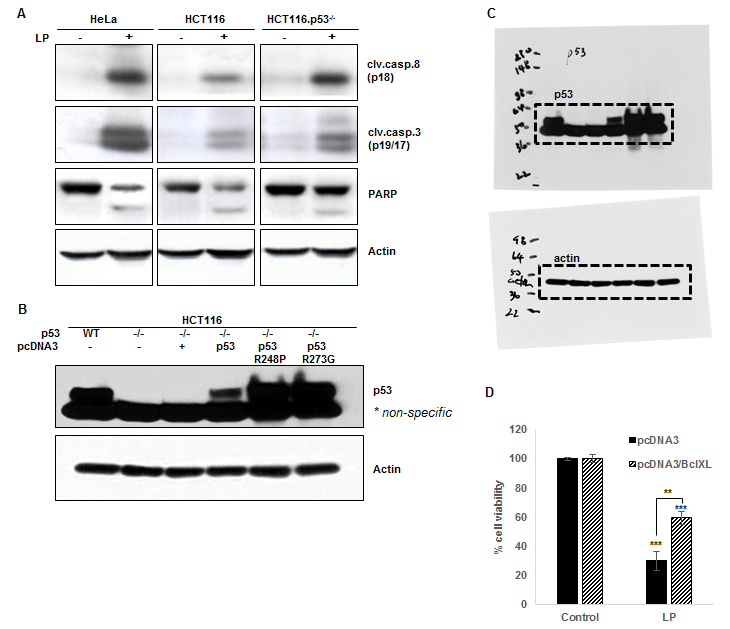


**Figure S3. Liquid plasma induces apoptotic cancer cell death in a p53-independent manner and overexpression of anti-apoptotic Bcl-xL abrogates the LP-induced cancer cell death.** (**A**) The proteins PARP and caspase 3 and 8 were cleaved in LP-treated HeLa, p53 wild-type HCT116 and p53 knock-out HCT116 cells. (**B**) p53 expression in p53 wild-type or p53 knock-out HCT116 with reconstituted p53 wild type or mutant types. (**C**) Full-length images of the immunoblots presented in (**B**). (**D**) One day after LP treatment of HeLa cells exogenously expressing Bcl-xL, cell viability was evaluated by live/dead assay. NS, not significant; **P* ≤ 0.05, ***P* ≤ 0.01, ****P* ≤ 0.001.

**
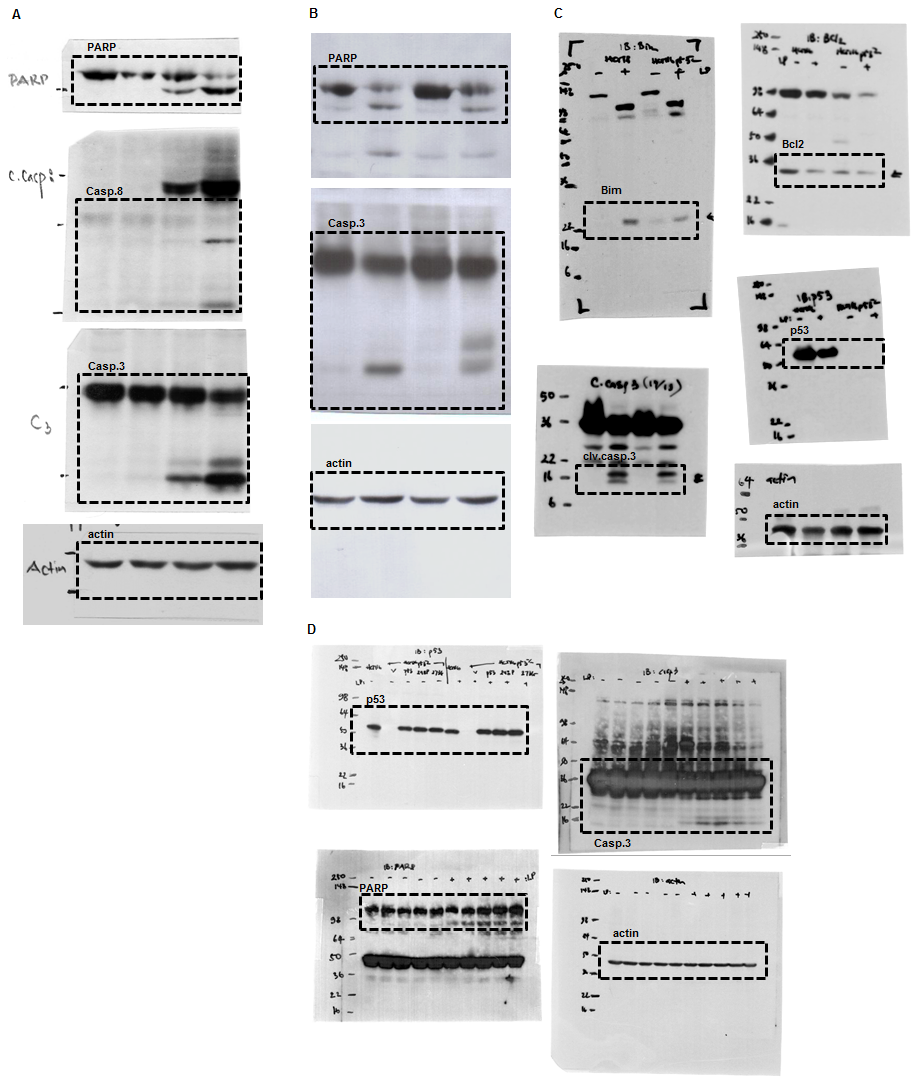
**

**Figure S4. Full-length images of the immunoblots presented in Figure 4.** The black dotted lines on the immunoblots indicate the cut outlines of the cropped images used in Figure 4. Full-length images of cropped immunoblots presented in Fig. 4B (in Fig. S4A), Fig. 4C (in Fig. S4B), Fig. 4D (in Fig. S4C) and Fig. 4F (in Fig. S4D) are shown.

**
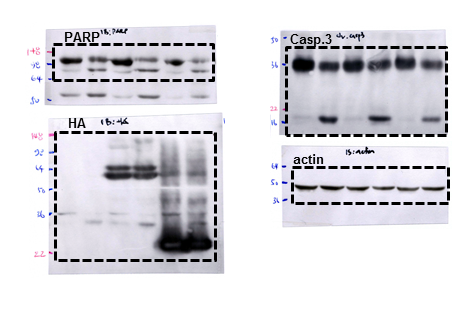
**

**Figure S5. Full-length images of the immunoblots presented in Figure 7C.** The black dotted lines on the immunoblots indicate the cut outlines of the cropped images used in Figure 7C.


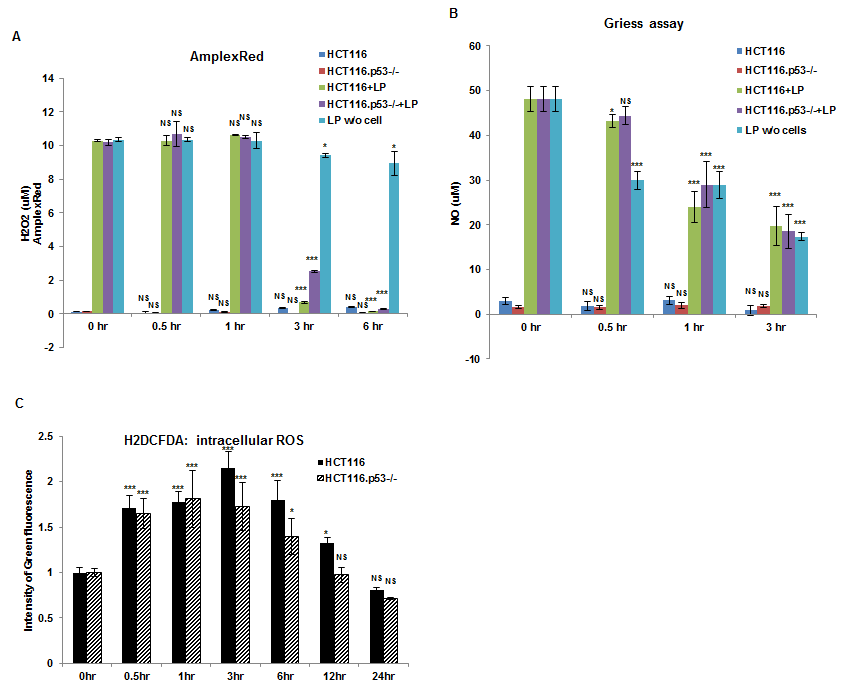


**Figure S6. Liquid plasma generated reactive oxygen and nitrogen species, and induced mitochondrial ROS accumulation in HCT116 and HCT116 p53 -/- cells**. Extra- and intracellular ROS and RNS were quantified by the AmplexRed assay (extracellular H_2_O_2_, (**S6A**)), the Griess assay (extracellular NO, (**S6B**)), and 5,6-carboxy-2′,7′-dichlorofluorescein diacetate (H2DCF-DA) assay (intracellular ROS, (**S6C**)). NS, not significant; **P* ≤ 0.05, ***P* ≤ 0.01, ****P* ≤ 0.001.
